# The genetic landscape of major drug metabolizing cytochrome P450 genes—an updated analysis of population-scale sequencing data

**DOI:** 10.1038/s41397-022-00288-2

**Published:** 2022-09-06

**Authors:** Yitian Zhou, Volker M. Lauschke

**Affiliations:** 1 Department of Physiology and Pharmacology, Karolinska Institutet, 171 77 Stockholm, Sweden; 2 Dr Margarete Fischer-Bosch Institute of Clinical Pharmacology, Stuttgart, Germany; 3 University of Tuebingen, Tuebingen, Germany

**Keywords:** Predictive markers, Genetic markers

## Abstract

Genes encoding cytochrome P450 enzymes (CYPs) are extremely polymorphic and multiple *CYP* variants constitute clinically relevant biomarkers for the guidance of drug selection and dosing. We previously reported the distribution of the most relevant *CYP* alleles using population-scale sequencing data. Here, we update these findings by making use of the increasing wealth of data, incorporating whole exome and whole genome sequencing data from 141,614 unrelated individuals across 12 human populations. We furthermore extend our previous studies by systematically considering also uncharacterized rare alleles and reveal that they contribute between 1.5% and 17.5% to the overall genetically encoded functional variability. By using established guidelines, we aggregate and translate the available sequencing data into population-specific patterns of metabolizer phenotypes. Combined, the presented data refine the worldwide landscape of ethnogeographic variability in *CYP* genes and aspire to provide a relevant resource for the optimization of population-specific genotyping strategies and precision public health.

## Introduction

Drug response can vary substantially between individuals with up to 50% of patients undergoing pharmacotherapy suffering from low treatment efficacy or adverse drug reactions [1, 2]. Overall, around 30% of this variability has been attributed to genetic factors and variations in cytochrome P450 (*CYP*) genes alone have been estimated to be relevant for 10–20% of all drug therapies [3]. Notably, of the 57 CYP enzymes encoded in the human genome, eight (CYP2A6, CYP2B6, CYP2C8, CYP2C9, CYP2C19, CYP2D6, CYP3A4 and CYP3A5) are responsible for the metabolism of most drugs in clinical use [4]. With the exception of CYP3A4, these enzymes lack important endogeneous substrates and, consequently, these *CYP* genes are extremely polymorphic with a plethora of single nucleotide variations (SNVs) and structural variants [5, 6]. Genetic drift, population admixture and isolation compound the genetic complexity and result in substantial ethnogeographic differences in *CYP* gene variability across human populations [7].

Besides common polymorphisms, recent advances in Next Generation Sequencing (NGS) have facilitated the identification of tens of thousands of rare variants across the human pharmacogenome [8–10]. However, the vast majority of studies to date only analyzed the frequency and distribution of common genetic candidate polymorphisms in *CYP* genes. We previously used exome sequencing data from 56,945 individuals to analyze the distribution of clinically relevant *CYP* star alleles across five major human populations [11]. Here, we update these data for 98 star alleles across the eight clinically most relevant *CYP* genes. Specifically, we analyzed fully consistent and compatible whole exome and whole genome sequencing data from 141,614 individuals and extend our analyses to twelve well-defined ethnogeographic groups. In addition, we comprehensively map and functionally interpret the rare genetic variability within these *CYP* genes and integrate both star alleles and uncharacterized variants to infer interethnic differences in phenotype distributions and human drug metabolism.

## Materials and methods

### Data sources

Star alleles of the analyzed *CYP* genes were defined based on PharmVar [12]. Variant frequency data from a total of 141,614 individuals were derived from the aggregated publicly available sequencing resource gnomAD [13]. Haplotype frequencies were derived considering population-specific linkage disequilibria between the respective polymorphisms based on data from the 1000 Genomes Project using LDlink [14]. For Ashkenazim and Koreans, linkage information from Europe and East Asia were used, respectively. As suballeles of a given star allele do not differ in allele function, they were aggregated throughout this study. Variant calls from short-read sequencing data can be problematic for *CYP2B6* [15] and *CYP2D6* [16], resulting in possible underestimations of variant frequency. To ameliorate these issues, we matched the extracted frequency information from gnomAD to data from the National Center for Biotechnology Information (NCBI) Allele Frequency Aggregator (ALFA), which includes sequencing data generated using longer reads. Notably, of all analyzed *CYP2B6* and *CYP2D6* variants, only rs2279343 was considerably underestimated in gnomAD (10.7% and 24.1% for Europeans and Africans in gnomAD vs 23.1% and 33.1% in ALFA, respectively; Supplementary Table 1). Thus, frequencies of the respective alleles were calculated using data from ALFA instead. Frequencies of the *1 reference allele (*f*_REF_) were calculated as *f*_REF_ = 1 − ∑_i_ *f*_i_, with *f*_i_ being the frequency of each considered variant allele *i*. Common and rare variants are defined as having minor allele frequencies (MAF) of ≥1% and <1%, respectively.

### Evaluation of variant functionality

The functional effects of star allele variants were obtained from the literature. For genetic variants for which effects have not been described, functional consequences were estimated using the ADME-optimized prediction framework (APF) [17]. In brief, APF generates an ensemble score for each variant by integrating five computational algorithms (LRT, MutationAssessor, PROVEAN, VEST3 and CADD) using parameter configurations specifically optimized for pharmacogenomic assessments. Notably, while APF is in principle also applicable to non-coding variations, it has not yet been benchmarked for this purpose and is thus applied here only to variants that affect the amino acid sequence of the respective gene product.

### Inferring phenotypes from functional variants

To infer phenotypes, we considered exonic variants with known or putative functional effects, copy number variations in *CYP2A6* and *CYP2D6*, as well as selected intronic (CYP3A4*22 and CYP3A5*3) and regulatory (CYP2A6*9, CYP2B6*22 and CYP2C19*17) variants outside of exons. All variations and haplotypes were assigned a functionality score based on the respective CPIC guidelines where available. For uncharacterized variations we used APF scores as activity score predictions. Both established (CPIC) and predicted (APF) activity scores of variants were then aggregated to infer metabolizer phenotype distributions for each population by calculating diplotype frequencies assuming Hardy-Weinberg equilibrium.

## Results

### Update on the global distribution of clinically relevant *CYP* alleles

**Fig. 1:**
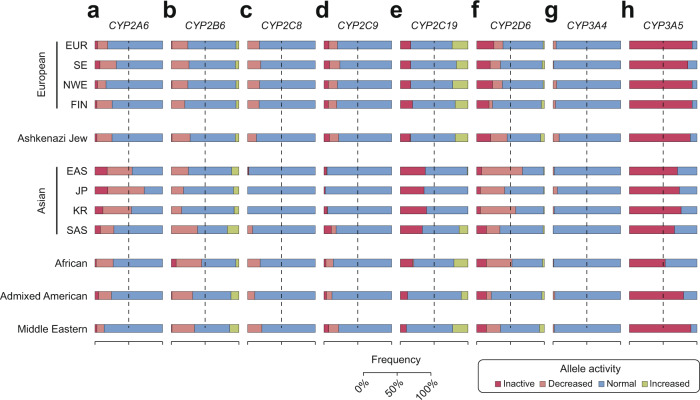
Ethnogeographic distribution of the inferred functional consequences of well-characterized *CYP* star alleles. Frequencies of inactive (dark red), reduced activity (light red), normal (blue) and increased activity (green) alleles shown in Tables 1–5 were aggregated for *CYP2A6* (**a**), *CYP2B6* (**b**), *CYP2C8* (**c**), *CYP2C9* (**d**), *CYP2C19* (**e**), *CYP2D6* (**f**), *CYP3A4* (**g**) and *CYP3A5* (**h**) genes across 12 populations. Star alleles with unclear functional consequences were considered as normal. EUR European, SE South European, NEW North-West European, FIN Finnish, EAS East Asian, JP Japanese, KR South Korean, SAS South Asian.

First, we analyzed the ethnogeographic distribution of a total of 98 well-characterized star alleles in eight *CYP* genes across 12 populations. Of the 16 alleles studied in *CYP2A6*, *2, *4 (gene deletion) and *5 abolish function, whereas *7, *9, *17, *18, *21, *23 and *35 constitute reduced function alleles (Table 1). Overall, East Asian populations harbor the highest frequency of inactive and decreased function alleles, primarily due to the high frequencies of *4, *7 and *9 (Fig. 1a). Particularly in Japanese, frequencies of these three alleles were highest among all populations with MAFs of 19%, 13.7% and 30%, respectively. In contrast, the loss-of-function allele CYP2A6*2 was not detected in East Asian populations, whereas it was common in European populations (MAF ≥ 2.5%). Within Europe, *CYP2A6* gene deletions were common in Southern Europeans (MAF = 4%), whereas frequencies were considerably lower throughout the rest of Europe (MAF ≤ 1%). Among the population-specific *CYP2A6* alleles, *23, *25 and *28 were exclusively found in Africans with MAFs pivoting around 1.4%, whereas *8 and *19 were only detected in Asia (MAF = 1.8–2.4% and 0.8–1.3%).

**Table 1:** Important variant and allele frequencies of the human *CYP2A6* gene.

Allele	Defining variants	Variant type	Functional consequence	European				AJ	Asian				AFR	AMR	ME
				Overall	SE	NWE	FIN		EAS	JP	KR	SAS			
*1	None		Normal	77	55	79.1	73.9	72.6	41.7	23.8	42.8	68.6	69.8	74.3	84.8
*2	rs1801272	Missense (L160H)	Inactive	2.6	2.8	2.8	2.5	2.2	0	0	0	1.2	0.4	1.6	1.3
*4	CYP2A6 deleted		Inactive	1	4	1	1	0	17	19	10.8	7	1.5	4	1
*5	rs5031017	Missense (G479V)	Inactive	<0.1	0	<0.1	0.1	<0.1	0.7	0	1	<0.1	<0.1	<0.1	0
*7	rs5031016	Missense (I471T)	Decreased	<0.1	0.1	<0.1	<0.1	0.5	8.9	13.7	12.5	0.1	<0.1	<0.1	0.8
*8	rs28399468	Missense (R485L)	Normal	<0.1	0	0	0	0	2.3	2.4	1.8	0	0	0	0
*9	rs28399433	TATA box	Decreased	6.8	5.5	6.3	11.2	7.1	22.5	30	24.1	14.2	8.3	14.1	6.1
*14	rs28399435	Missense (S29N)		4.1	3.1	4.5	1.3	2.1	<0.1	0	0	3.6	0.8	1.5	1.6
*17	rs28399454	Missense (V365M)	Decreased	<0.1	<0.1	<0.1	0	<0.1	0	0	0	<0.1	10.9	0.6	0.9
*18	rs1809810	Missense (Y392F)	Decreased	1.4	1.3	1.6	1.4	0.7	0.3	0.8	0	1.2	0.5	1	1.5
*19	rs5031016, rs1809810	Missense (I471T, Y392F)		<0.1	0.1	<0.1	<0.1	0.2	0.8	1.3	1.2	<0.1	<0.1	<0.1	0.4
*21	rs6413474	Missense (K476R)	Decreased	1.3	1.1	1.3	2.6	2.9	<0.1	0	0	1.9	0.2	0.5	0.9
*23	rs56256500	Missense (R203C)	Decreased	<0.1	<0.1	<0.1	0	0	0	0	0	<0.1	1.4	<0.1	0
*25	rs28399440	Missense (F118L)		<0.1	0	<0.1	0	0	0	0	0	0	1.4	<0.1	0
*28	rs28399463, rs8192730	Missense (N418D, E419D)		<0.1	<0.1	<0.1	<0.1	0	<0.1	<0.1	<0.1	<0.1	1.5	<0.1	0
*35	rs143731390	Missense (N438Y)	Decreased	5.7	7.1	3.3	7	11.7	5.7	9.1	5.7	2.1	3.3	2	0.6

*AFR* African, *EAS* East Asian, *SAS* South Asian, *AMR* admixed Americans, *FIN* Finnish, *AJ* Ashkenazi Jewish, *ME* Middle Eastern, *SE* South European, *NWE* Northwest European, *JP* Japanese, *KR* Korean.

In contrast to *CYP2A6* which harbors multiple common loss-of-function alleles, the only common inactive *CYP2B6* allele is CYP2B6*18 in Africa (MAF = 7%; Table 2). The other *CYP2B6* loss-of-function alleles *8, *12, *13 and *28 are rare in all populations studied. Notably, the decreased function allele CYP2B6*6 (MAF = 14.5–32%) and the increased function allele CYP2B6*4 (MAF = 1–15.3%) are common across all ethnogeographic groups studied. Furthermore, CYP2B6*9 is common in most populations with MAFs between 2.5% and 11.1% with the exception of Finnish (MAF = 0%) and Koreans (MAF = 0.1%). Overall, inactive and reduced function *CYP2B6* alleles are most prevalent in African and South Asian populations, whereas frequencies are lowest in Koreans and Japanese (Fig. 1b).

**Table 2:** Important variant and allele frequencies of the human *CYP2B6* gene.

Allele	Defining variants		Variant type	Functional consequence	European				AJ	Asian				AFR	AMR	ME
					Overall	SE	NWE	FIN		EAS	JP	KR	SAS			
*1		None		Normal	55.7	54.4	55.8	62.1	50.8	74.4	74.4	80.7	47.8	43.6	57.3	47.1
*2		rs8192709	Missense (R22C)	Normal	5.6	7.3	4.8	4.9	8	4.6	4.6	2.9	3.1	3.7	3.1	10.1
*4		rs2279343	Missense (K262R)	Increased	3.4	3.4	3.4	3.4	3.4	10.6	7.7	7.2	15.3	1	10.2	10.4
*5		rs3211371	Missense (R487C)	Normal	11.8	10.3	11.9	12.1	10.4	0	1.6	0.2	7.5	2.6	4.4	6.8
*6		rs2279343, rs3745274	Missense (K262R, Q172H)	Decreased	19.6	19.7	19.3	19.7	19.4	21.5	15.5	14.5	28.1	32	20.3	22.8
*7		rs2279343, rs3745274, rs3211371	Missense (K262R, Q172H, R487C)	Decreased	<0.1	0	0.4	0	0.3	0.2	0.2	0.3	0.1	0	0	0.3
*8		rs12721655	Missense (K139E)	Inactive	0.4	0.2	0.5	<0.1	0	0	0	0	0	<0.1	0.1	0
*9		rs3745274	Missense (Q172H)	Decreased	4.4	5.8	4.5	0	7.2	3.4	2.5	0.1	10.3	5.1	11.1	10.2
*12		rs36060847	Missense (G99E)	Inactive	<0.1	<0.1	<0.1	0	0.7	0	0	0	0	<0.1	<0.1	0
*13		rs2279343, rs3745274, rs12721655	Missense (K262R, Q172H, K139E)	Inactive	0	0	0	0	0	0	0	0	<0.1	0	0	0
*18		rs28399499	Missense (I328T)	Inactive	<0.1	<0.1	<0.1	0	0	0	0	0	<0.1	7	0.4	0.6
*19		rs34826503	Missense (R336C)	Decreased	<0.1	<0.1	0	0	0	<0.1	0	<0.1	0	0.2	<0.1	0
*20		rs36056539	Missense (T168I)	Decreased	0	0	0	0	0	0	0	0	0	0.2	<0.1	0
*22		rs34223104	Regulatory	Increased	1.3	1.9	1	0.7	1.7	0.3	0	0	1.8	3	1.4	3.2
*26		rs2279343, rs3745274, rs3826711	Missense (K262R, Q172H, P167A)	Decreased	0	0	0	0	0	0.4	0	0.3	0	0	0	0
*28		rs34097093	Stop-gain (R378X)	Inactive	<0.1	<0.1	<0.1	<0.1	0	0	0	0	0	<0.1	<0.1	0

*AFR* African, *EAS* East Asian, *SAS* South Asian, *AMR* admixed Americans, *FIN* Finnish, *AJ* Ashkenazi Jewish, *ME* Middle Eastern, *SE* South European, *NWE* Northwest European, *JP* Japanese, *KR* Korean.

For *CYP2C8*, the genetic variability was overall considerably lower than for *CYP2A6* and *CYP2B6*. European, Middle Eastern and African populations feature similar frequency of decreased function *CYP2C8* alleles (Fig. 1c). In Africans, the major allele is CYP2C8*2 (MAF = 15.2%), whereas *3 (MAF = 2–15.2%) and *4 (MAF = 1.8–5.8%) are the primary minor alleles in the other populations (Table 3). In contrast, *CYP2C8* is extremely conserved in East Asian populations with >99.4% of all alleles corresponding to the reference sequence.

**Table 3:** Important variant and allele frequencies of the human *CYP2C* gene family.

Allele	Defining variants	Variant type	Functional consequence	European				AJ	Asian				AFR	AMR	ME
				Overall	SE	NWE	FIN		EAS	JP	KR	SAS			
*CYP2C8*															
*1	None		Normal	82.9	81	82.6	83.1	86.9	99.3	100	100	92.5	81.6	89.7	78.8
*2	rs11572103	Missense (I269F)	Decreased	0.3	0.6	0.2	<0.1	1.4	<0.1	0	0	1.9	15.2	0.8	3.5
*3	rs10509681, rs11572080	Missense (K399R, R139K)	Controversial	11.3	13.2	11.8	11.1	9.9	<0.1	0	0	4.1	2	6.8	15.2
*4	rs1058930	Missense (I264M)	Decreased	5.4	5.2	5.3	5.8	1.8	<0.1	0	0	1.5	1.1	2.7	2.2
*5	rs72558196	Frameshift	Inactive	0	0	0	0	0	0.2	0	0	0	0	0	0
*7	rs72558195	Stop-gain (R186X)	Inactive	<0.1	<0.1	<0.1	<0.1	0	<0.1	0	0	<0.1	<0.1	<0.1	0.3
*11	rs78637571	Stop-gain (E274X)	Inactive	0	0	0	0	0	0.4	0	0	0	0	<0.1	0
*CYP2C9*															
*1	None		Normal	79.9	76.7	79.7	81.5	78	95.9	98	94.9	82.1	78.9	88.4	77.5
*2	rs1799853	Missense (R144C)	Decreased	12.6	14.2	13.1	11.4	13.5	<0.1	0	<0.1	4.7	2.2	6.8	13.6
*3	rs1057910	Missense (I359L)	Inactive	6.8	8.5	6.5	6.3	8.4	3.3	0.7	4.4	11	1.2	3.8	7.3
*5	rs28371686	Missense (D360E)	Decreased	<0.1	<0.1	<0.1	0	0	0	0	0	<0.1	1.1	<0.1	0
*6	rs9332131	Frameshift	Inactive	<0.1	<0.1	<0.1	0	0	0	0	0	0	1	<0.1	0
*8	rs7900194	Missense (R150H)	Decreased	<0.1	<0.1	<0.1	0	0	<0.1	0	0	<0.1	6	0.2	0
*9	rs2256871	Missense (H251R)	Normal	<0.1	<0.1	<0.1	0	0	<0.1	0	0	<0.1	7.5	0.3	0.9
*11	rs28371685	Missense (R335W)	Decreased	0.3	0.2	0.3	0.6	0	<0.1	0	0	0.2	1.9	0.2	0.6
*12	rs9332239	Missense (P489S)	Decreased	0.3	0.3	0.2	0.2	<0.1	0	0	0	<0.1	<0.1	0.1	0
*13	rs72558187	Missense (L90P)	Inactive	0	0	0	0	0	0.2	0.7	0.2	0	0	0	0
*14	rs72558189	Missense (R125H)	Decreased	<0.1	<0.1	<0.1	0	<0.1	<0.1	0	<0.1	1.9	0	<0.1	0
*16	rs72558192	Missense (T299A)	Decreased	<0.1	0	0	0	0	0.4	0	0	0	0	0	0
*29	rs182132442	Missense (P279T)	Decreased	<0.1	<0.1	<0.1	<0.1	0	0.1	0	0.4	<0.1	<0.1	<0.1	0
*31	rs57505750	Missense (I327T)	Decreased	0	0	0	0	0	<0.1	0.7	0	0	<0.1	<0.1	0
*CYP2C19*															
*1	None		Normal	61.5	67.6	62.5	63.6	65.7	62.1	64.8	61.7	67.1	55.8	79.1	68.2
*2	rs4244285	Splicing defect	Inactive	14.7	14.7	14.8	17.5	13.2	30.8	26.7	28	32.4	17.8	10.1	8.7
*3	rs4986893	Stop-gain (W212X)	Inactive	<0.1	<0.1	<0.1	<0.1	0	6.3	8.6	10.2	0.4	<0.1	<0.1	0
*4	rs28399504	Start lost	Inactive	0.3	0.4	0.3	0	1.6	<0.1	0	<0.1	<0.1	<0.1	0.3	0
*5	rs56337013	Missense (R433W)	Inactive	<0.1	<0.1	0	0	0	<0.1	0	<0.1	<0.1	0	0	0
*6	rs72552267	Missense (R132Q)	Inactive	<0.1	<0.1	<0.1	0	0	<0.1	0	0	0	<0.1	<0.1	0
*7	rs72558186	Splicing defect	Inactive	0	0	0	0	0	0	0	0	<0.1	0	0	NA
*8	rs41291556	Missense (W120R)	Inactive	0.3	0.2	0.3	<0.1	<0.1	0	0	0	<0.1	<0.1	<0.1	0
*9	rs17884712	Missense (R144H)	Decreased	<0.1	<0.1	<0.1	0	<0.1	0	0	0	<0.1	1.3	<0.1	0
*10	rs6413438	Missense (P227L)	Decreased	<0.1	0	<0.1	0	0.1	<0.1	0	0	0	0.3	<0.1	0
*13	rs17879685	Missense (R410C)	Normal	<0.1	0	<0.1	0	0	0	0	0	<0.1	1.8	<0.1	0
*15	rs17882687	Missense (I19L)	Normal	<0.1	<0.1	<0.1	0	0.4	0	0	0	<0.1	1.9	<0.1	0.3
*17	rs12248560	Regulatory	Increased	23.1	17	22	18.8	19	0.7	0	0	13.6	20.9	10.1	22.8
*22	rs140278421	Missense (R186P)	Inactive	<0.1	0	0	0	0	0	0	0	0	0.1	<0.1	0

*AFR* African, *EAS* East Asian, *SAS* South Asian, *AMR* admixed Americans, *FIN* Finnish, *AJ* Ashkenazi Jewish, *ME* Middle Eastern, *SE* South European, *NWE* Northwest European, *JP* Japanese, *KR* Korean.

Similar patterns with overall low variability in East Asian populations are observed for *CYP2C9* (Fig. 1d), consistent with previous reports of high linkage between *CYP2C8* and *CYP2C9* haplotypes [18]. The reduced function allele CYP2C9*2 was globally common (MAF = 2.2–14.2%) with the exception of East Asian populations (MAF < 0.1%; Table 3). *CYP2C9* furthermore harbors various population-specific alleles. CYP2C9*6 (MAF = 1%), *8 (MAF = 6%), *9 (MAF = 7.5%) and *11 (MAF = 1.9%) are only common in Africans, while *13 (MAF = 0.7%), *16 (MAF = 0.4%), *29 (MAF = 0.4%) and *31 (MAF = 0.7%) are only found in Asian populations, albeit at lower frequencies.

In *CYP2C19*, only the splice site loss-of-function allele CYP2C19*2 is common in all populations analyzed (MAF = 8.7–32.4%; Table 3). The regulatory increased function allele CYP2C19*17 is frequent in European, admixed American, Middle Eastern and African populations with MAFs between 10.1% and 23.1%, whereas it is rare across East Asia (MAF = 0–0.7%). Notable among the population-specific variations are the loss-of-function variants CYP2C19*3 and *4 as well as the decreased function variant *9, which are exclusively found in East Asian (MAF ≥ 6.3%), Ashkenazim (MAF = 1.6%) and African populations (MAF = 1.3%), respectively. Overall, unlike for the other *CYP2C* genes, *CYP2C19* loss-of-function alleles are most common and increased function alleles are most rare in East Asians, whereas patterns of allele activity are very similar across all other analyzed populations (Fig. 1e).

*CYP2D6* constitutes the most polymorphic pharmacogene and harbors a multitude of common variants with clinical relevance, particularly for the treatment with antidepressants, antipsychotics, opioid analgesics and antihypertensives. Here, we studied 17 *CYP2D6* alleles, 13 of which are associated with altered allele function. Decreased function alleles are most prevalent in East Asians due to high frequencies of CYP2D6*10 (MAF ≥ 35%), whereas loss-of-function alleles are most abundant in European populations primarily because of high frequencies of the splicing variant CYP2D6*4 (MAF up to 20.3%) (Table 4 and Fig. 1f). Notably, reduced *CYP2D6* allele function are also very common in Africans; however, in these populations the primary drivers are *17 (MAF = 20.5%) and *29 (MAF = 8.9%), which are almost exclusively found in this population. Increased function due to the functional gene duplications CYP2D6*1xN and *2xN is most common in Middle Eastern populations (aggregated MAF = 7%), Ashkenazi Jews (aggregated MAF = 5.6%) and Europeans (aggregated MAF up to 4.7%) but were rare in East Asian populations (aggregated MAF < 1%).

**Table 4:** Important variant and allele frequencies of the human *CYP2D6* gene.

Allele	Defining variants	Variant type	Functional consequence	European				AJ	Asian				AFR	AMR	ME
				EUR	SE	NWE	FIN		EAS	JP	KR	SAS			
*1	None		Normal	24.1	27.3	27.7	31.8	7.9	17.5	42	27.9	25.3	20.8	48.9	6.7
*1xN	Amplification of *1		Increased	0.9	1.6	1	2.5	3	0.3	0.5	<0.1	0.6	1.5	1.1	3.1
*2	rs16947, rs1135840	Missense (R296C, S486T)	Normal	33.6	37.2	33.5	39.1	40.4	14.6	15.8	13.6	38	22.5	26	50.6
*2xN	Amplification of *2		Increased	1.2	1.2	0.9	2.2	2.6	0.4	0.4	0.8	1.1	1.3	1.9	3.9
*3	rs35742686	Frameshift	Inactive	1.7	1.1	1.7	3.6	0.4	0	0	0	0.1	0.3	0.5	0.3
*4	rs3892097	Splicing defect	Inactive	19.6	17.1	20.3	10	18.2	0.3	0	0.2	10.4	8	11.1	9.8
*5	CYP2D6 deleted		Inactive	2.9	2.3	4.1	2.2	1.1	5.2	4.9	4.9	3.2	6.2	2.1	2.3
*6	rs5030655	Frameshift	Inactive	1.1	1.1	1.2	2.1	0.8	0	0	0	0.2	0.2	0.4	0.6
*7	rs5030867	Missense (H324P)	Inactive	<0.1	<0.1	<0.1	0	<0.1	0	0	0	0.8	<0.1	<0.1	1.3
*9	rs5030656	Inframe deletion (K281del)	Decreased	2.6	1.9	3	1.3	0.3	0	0	0	0.2	0.4	1.2	0
*10	rs1065852, rs1135840	Missense (P34S, S486T)	Decreased	1.7	2.4	1.5	1.1	6.7	57.3	35	50.2	5.5	4.8	1.4	6.4
*14	rs5030865	Missense (G169R)	Decreased	<0.1	0	0	0	0	1.3	0.8	0.5	<0.1	0	<0.1	0
*17	rs16947, rs28371706	Missense (R296C, T107I)	Decreased	<0.1	0.4	0	0	0	0	0	0	0	20.5	0.7	0
*29	rs16947, rs1135840, rs61736512, rs59421388	Missense (R296C, S486T, V136I, V338M)	Decreased	0	0	0	0	0	0	0	0	0	8.9	0.4	0
*41	rs28371725	Splicing defect	Decreased	9.3	10.9	10	3.2	17.6	3.2	0.7	1.8	13.6	2.6	4	14.3
*33	rs28371717	Missense (A237S)	Normal	1.2	0.5	1.1	1	1	0	0	0	0.7	0.2	0.2	0.6
*43	rs28371696	Missense (R26H)		<0.1	<0.1	<0.1	<0.1	0	<0.1	0	<0.1	0.6	1.8	0.2	0

*AFR* African, *EAS* East Asian, *SAS* South Asian, *AMR* admixed Americans, *FIN* Finnish, *AJ* Ashkenazi Jewish, *ME* Middle Eastern, *SE* South European, *NWE* Northwest European, *JP* Japanese, *KR* Korean.

Among the major drug metabolizing CYPs, CYP3A4 is the only enzyme with an important endogenous substrate and, as a consequence, *CYP3A4* is the most conserved among the studied *CYPs* (Table 5 and Fig. 1g). In Europeans, admixed Americans, Ashkenazi Jews and Middle Easterners, CYP3A4*22 is the only common allele of functional relevance with frequencies ranging between 0.9% in South Europeans to 9% in Ashkenazim. In contrast, in East Asian populations the population-specific *16 and *18 alleles are common, the former of which only in Japanese (MAF = 3.9%), whereas *22 is absent. Notably, South Asian populations do not harbor common *CYP3A4* alleles.

**Table 5:** Important variant and allele frequencies of the human *CYP3A* gene family.

Allele	Defining variants	Variant type	Functional consequence	European				AJ	Asian				AFR	AMR	ME
				Overall	SE	NWE	FIN		EAS	JP	KR	SAS			
*CYP3A4*															
*1	None		Normal	94.7	98.4	93.9	93.4	91	97.5	93.4	98.2	99.4	96.2	97.1	98.1
*2	rs55785340	Missense (S222P)		<0.1	0	<0.1	1	0	0	0	0	0	0	0	0
*3	rs4986910	Missense (M445T)		0.7	0.6	0.7	1.8	<0.1	0	0	0	<0.1	<0.1	0.2	0
*4	rs55951658	Missense (I118V)		0	0	0	0	0	0.5	0	0.2	<0.1	0	<0.1	0
*8	rs72552799	Missense (R130Q)	Decreased	0.1	<0.1	<0.1	0.2	0	0	0	0	<0.1	<0.1	<0.1	0
*15	rs4986907	Missense (R162Q)		<0.1	<0.1	<0.1	0	<0.1	0	0	0	<0.1	2.6	0.2	0.3
*16	rs12721627	Missense (T185S)	Decreased	0	0	0	0	0	<0.1	3.9	0.3	0	0	0	0
*18	rs28371759	Missense (L293P)	Decreased	0	0	0	0	0	1.9	2.6	1.4	<0.1	0.1	<0.1	0
*22	rs35599367	Splicing defect	Decreased	4.4	0.9	5.4	3.6	9	0	0	0	0.6	0.9	2.5	1.6
*CYP3A5*															
*1	None		Normal	6.4	12.6	6.4	6.8	9.9	28.6	26	23.5	33.2	47	19.5	8.5
*2	rs28365083	Missense (T398N)		0.6	0.4	0.6	0.2	<0.1	0	0	0	0	0.1	0.1	0
*3	rs776746	Splicing defect	Inactive	92.9	86.8	92.9	93	90	71.4	74	76.5	66.8	29.8	79.2	88
*6	rs10264272	Splicing defect	Inactive	<0.1	0.2	<0.1	<0.1	0	0	0	0	<0.1	12.9	0.8	3.5
*7	rs41303343	Frameshift	Inactive	<0.1	<0.1	<0.1	0	0	<0.1	0	0	<0.1	10.2	0.5	0

*AFR* African, *EAS* East Asian, *SAS* South Asian, *AMR* admixed Americans, *FIN* Finnish, *AJ* Ashkenazi Jewish, *ME* Middle Eastern, *SE* South European, *NWE* Northwest European, *JP* Japanese, *KR* Korean.

CYP3A5 activity is primarily governed by the CYP3A5*3 allele, which is defined by the presence of a variant that results in the generation of a cryptic splice site that causes a premature stop codon. The *3 allele constitutes the major allele in most populations studied (MAF = 66.8–93%) except for Africans (MAF = 29.8%; Table 5). The latter also harbor the population-specific loss-of-function frameshift allele CYP3A5*7 (MAF = 10.2%) as well as CYP3A5*6 (MAF = 12.9%), a splice variant restricted to African and Middle Eastern populations. Consequently, 53% to 94% of all *CYP3A5* alleles are inactive across all major populations (Fig. 1h).

### The genetic landscape of *CYP* variability

**Fig. 2:**
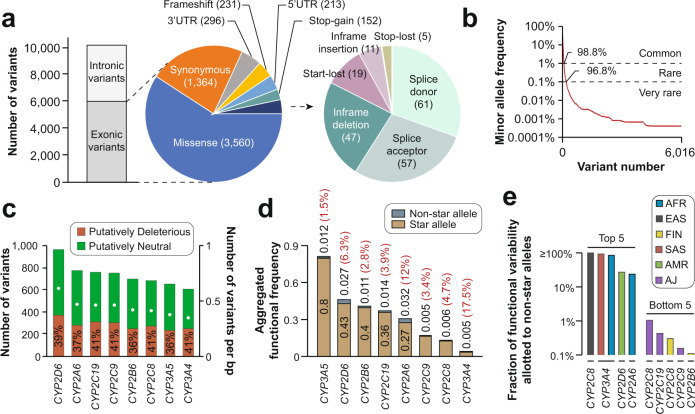
The landscape of genetic variability in major drug-metabolizing CYP genes. **a** Across 141,614 individuals, a total of 10,176 genetic variants were identified 6016 of which were exonic. Pie charts indicate the distribution of exonic variants across variant classes. **b** The majority of exonic variants have MAFs < 1% (98.8%) or <0.1% (96.8%). **c** Stacked column plot showing the number of identified exonic variants for each analyzed *CYP* gene (left *y*-axis). White dots indicate the number of variants per base pair (bp; right *y*-axis). All variants were categorized into deleterious (red) or neutral (green) using the ADME Prediction Framework (see Methods). The fraction of putatively deleterious variants (including both reduced function and loss-of-function variants) for each *CYP* gene is shown. A list of all putatively deleterious variants and their estimated activity scores is provided in Supplementary Table 2. **d** Functional contribution of star alleles and non-star alleles to the overall functional variability of *CYP* genes. Black numbers indicate the aggregated frequencies of functional star alleles and non-star alleles. The ratio of the genetically encoded functional variability allotted to non-star alleles and star alleles is indicated by the red percentage values. **e** Functional contribution of non-star alleles to the population-specific functional variability of *CYP* genes. Only the top 5 and bottom 5 gene-population pairs are shown. All population-specific contributions of non-star alleles are shown in Supplementary Table 3. AFR African, EAS East Asian, SAS South Asian, AMR admixed Americans, FIN Finnish, AJ Ashkenazi Jewish, MAF minor allele frequency, UTR untranslated region.

In addition to the well-characterized star alleles, *CYP* genes harbor a multitude of variants with unknown functional consequences. Using whole exome and whole genome sequencing data from 141,614 unrelated individuals, a total of 10,176 genetic variants were identified across the eight *CYPs*, of which 6016 were exonic (Fig. 2a). Notably, intronic variations are likely underreported as the majority of samples were sequencing using exome sequencing, which does not systematically cover introns. We thus we focused our further analyses exclusively on exonic variants. Among the exonic variations, missense (*n* = 3560; 59%) and synonymous variants (*n* = 1364; 23%) were most common, whereas only 19 start-lost variants, 11 in-frame insertions and 5 stop lost variants were identified. Importantly, 98.8% (*n* = 5891) and 96.8% (*n* = 5695) of the exonic variants were rare with MAFs < 1% and MAF < 0.1%, respectively (Fig. 2b). Compared to the approximately 150 variants with established functional annotations based on experimental or epidemiological data, these results indicate that the functional impact of the vast majority of variants in drug metabolizing *CYPs* remains to be determined.

To estimate the functional relevance of these unexplored variations at the population scale, we thus utilized computational predictions. Specifically, we used the APF algorithm (see Methods), which has been specifically developed for the interpretation of genetic variability in pharmacogenes. Overall, we identified 2175 variants that were predicted to reduce enzyme function (Supplementary Table 2). While more variants were identified in *CYP2D6* (*n* = 962; 0.65 per bp), *CYP2A6* (*n* = 768; 0.52 per bp) and *CYP2C19* (*n* = 753; 0.51 per bp) and less in *CYP2C8* (*n* = 679; 0.46 per bp), *CYP3A5* (*n* = 654; 0.43 per bp) and *CYP3A4* (*n* = 609; 0.4 per bp), the fraction of variants with impacts on gene function was similar across all *CYPs* (36–41%; Fig. 2c). Next, we aggregated the functional predictions based on computational inference with the functional variability allotted to star alleles (Fig. 2d). Expectedly, *CYP3A5* harbored the overall largest fraction of altered function alleles (81.2%), followed by *CYP2D6* (45.7%) and *CYP2B6* (41.1%) and lowest was in *CYP2C9* (16.7%), *CYP2C8* (13.8%) and *CYP3A4* (3.7%). Globally, star alleles accounted for majority of functionally relevant alleles with non-star alleles contributing between 1.5% for *CYP3A5* and 17.5% in *CYP3A4* to the total genetically encoded functional variability. However, when stratifying the analysis by population, cases were observed where the putative functional impact of non-star alleles was considerable (Fig. 2e and Supplementary Table 3). In East Asian populations, uncharacterized variants in *CYP2C8* are predicted to be as relevant as the deleterious *CYP2C8* star alleles *2, *3, *4, *5, *7 and *11. Similarly, non-star alleles in *CYP3A4* were estimated to have similar impacts compared to star alleles in South Asian and African populations, and more than one fifth of the functional variability of *CYP2D6* and *CYPA6* can be attributed to non-star alleles in admixed Americans and Africans, respectively. In contrast, the functional contribution from non-star alleles in Ashkenazi Jews and Finnish are found to be marginal (Fig. 2e).

### Translation of *CYP* genetic variability profiles into population-specific functional effects

**Fig. 3:**
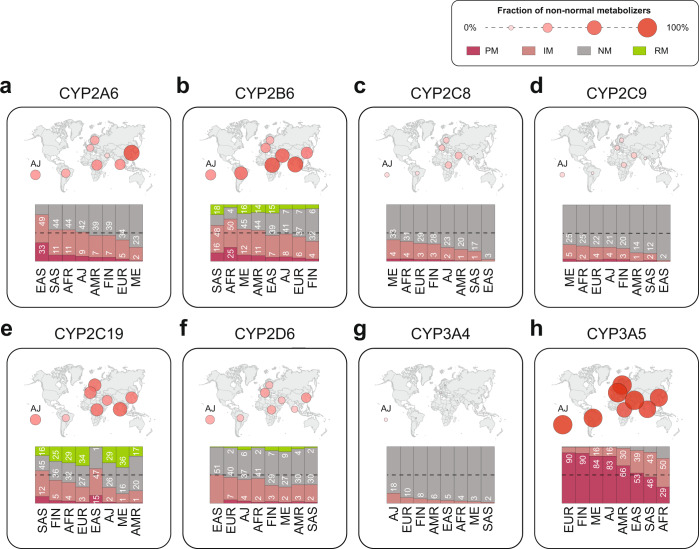
Global distributions of metabolic phenotypes. Frequencies of inferred population-specific metabolic phenotypes are shown for *CYP2A6* (**a**), *CYP2B6* (**b**), *CYP2C8* (**c**), *CYP2C9* (**d**), *CYP2C19* (**e**), *CYP2D6* (**f**), *CYP3A4* (**g**) and *CYP3A5* (**h**). Numbers indicate the fraction of poor metabolizers (PM; dark red), intermediate metabolizers (IM; light red) and rapid metabolizers (RM; green). The fraction of non-normal metabolizers is indicated by the size of circles on the respective world maps. AFR African, EAS East Asian, SAS South Asian, AMR admixed Americans, FIN Finnish, AJ Ashkenazi Jewish, ME Middle Eastern, EUR non-Finnish European.

Lastly, we used the aggregated inferred functionality data of star alleles and predicted uncharacterized variants to calculate the distributions of population-specific metabolizer phenotypes (Fig. 3). Poor metabolizers (PM) and intermediate metabolizers (IM) of CYP2A6 were most common in East Asian populations (33% PM; 49% IM). In contrast, only 5% and 2% of the population were CYP2A6 PMs in Europe and the Middle East (Fig. 3a). For CYP2B6, non-normal metabolizer phenotypes were most frequent in South Asia and Africa where only 19% and 21% were classified as normalizer metabolizers (NM; Fig. 3b). By contrast, 58%, 49% and 45% of Finnish, non-Finish European and Ashkenazim individuals were inferred to be NM. Moreover, rapid metabolizers (RM) for CYP2B6 were substantially more frequent in Asian and Middle Eastern populations as well as in admixed Americans (14–18% compared to ≤7% in other populations).

In contrast to CYP2A6 and CYP2B6, the majority of CYP2C8 and CYP2C9 classified as NM in every population analyzed (Fig. 3c, d). Impaired activity (IM or PM) of CYP2C8 and CYP2C9 was most prevalent in and Middle Easterners (38% and 30%, respectively) and Africans (35% and 27%, respectively), whereas East Asians are the most conserved among all studied populations (≤3% for both enzymes). In these calculations we considered CYP2C8*3 as a decreased function allele; however, as discussed below, functional effects of this allele are not clear and might be substrate-specific. An alternative map in which CYP2C8*2 is considered as functionally neutral is provided in Supplementary Fig. 1. For CYP2C19, South Asians were estimated to be phenotypically most variable, with 12%, 45% and 16% being classified as PM, IM and RM, respectively. In East Asians the fraction of PM (15%) and IM (47%) individuals were even higher than South Asians. However, only 1% of East Asians were CYP2C19 RM, whereas the respective frequencies in Middle Easterners (36%), Europeans (34%), Africans (29%) and Ashkenazim (29%) was substantially higher (Fig. 3e).

While *CYP2D6* has a multitude of population-specific polymorphisms with functional relevance, the fraction of non-normal CYP2D6 metabolizer phenotypes does not vary substantially across different ethnogeographic groups (Fig. 3f). The fraction of CYP2D6 IMs is highest in East Asians (51% of the population), whereas PMs are very rare (<1%). The highest frequency of CYP2D6 PMs is found in Europeans (7%) and Ashkenazim (4%). Normal CYP2D6 metabolizers are most common among individuals of Southeast Asian (65%) and admixed American (63%) descent while RMs are most frequent in the Middle East (9%).

For CYP3A4, the frequency of IMs is highest in Ashkenazi Jews and Europeans with an estimated 18% and 10% of the entire population, whereas the prevalence of IMs in other populations is <10% (Fig. 3g). In contrast, the majority of individuals across all populations are classified as IMs (9–50%) or PMs (29–90%; Fig. 3h). When using the older but still widely used classification scheme of expressors (defined as individuals with at least one active *CYP3A5* allele) and non-expressors (defined as individuals with two inactive *CYP3A5* alleles), the range of expressors varied between 10% in Europeans and 71% in Africans.

## Discussion

*CYP* genes are long known to be highly polymorphic with distinct genetic population differences. Comprehensive maps of interethnic differences in *CYP* variability have previously been presented for individual genes or alleles based on literature analyses [19–25]. Importantly however, genotyping strategies can differ between studies, which can impact allele frequency estimates particularly for genes with complex haplotype structures, such as *CYP2B6* and *CYP2D6*. In addition, there have been multiple efforts in mapping *CYP* variability by genotyping individuals across populations using consistent profiling approaches [26, 27]. However, due to practical limitations, these efforts were limited to relatively small cohorts. By analyzing comprehensive sequencing data from 141,614 individuals across a total of 12 populations we here update our previous meta-analysis [11] and provide a systematic overview of the global landscape of genetic variability for the eight *CYP* genes of highest clinical relevance.

Among the well-characterized *CYP* alleles, we find considerable differences between related ethnogeographic groups. In East Asia, despite substantial admixture of Japanese, Korean and Han Chinese [28], frequencies of functionally relevant alleles can differ substantially. For instance, the reduced function allele CYP3A4*16 is common in Japanese (MAF = 3.9%), whereas it is almost absent in Koreans and other East Asian populations with important implications for the treatment with statins and immunosuppressants. Furthermore, frequencies of CYP2D6*10 and CYP2D6*41 were substantially lower in Japanese compared to other East Asian groups, suggesting that recognition of these differences might aid in the optimization of population-specific dosing recommendations for antipsychotic and antidepressant treatment [29]. Our results moreover confirm considerable differences between Finnish and other European populations. Specifically, we find substantially lower frequencies of the reduced function alleles CYP2D6*41 (3.2% in Finnish compared to 9.3% in other European populations) as well as a lack of CYP2B6*9 (0% vs. 4.4%).

The functional effects of some common alleles remain controversial. For instance, CYP2C8*3, the most frequent *CYP2C8* variant allele in most populations, has been shown in vitro studies to result in reduced [30, 31], normal [32] or increased metabolism [33, 34]. Further complication is added by the fact that CYP2C8*3 is in strong linkage disequilibrium with the decreased activity allele CYP2C9*2 [35]. As both enzymes substantially overlap in their substrate specificity, this might have influenced previous clinical investigations in their interpretation that CYP2C8*3 results in decreased CYP2C8 activity [36].

Of note, phenotype assignments based on diplotypes differs between genes. For instance, for CYP2C9 NMs are defined as an activity score of 2 (i.e. individuals having two functional alleles), whereas IM status is assigned for individuals with activity scores of 1–1.5 and PMs are defined as 0–0.5 [37]. Similarly, an individual carrying a reduced function allele would be classified as IM for CYP2B6 [38] and CYP2C19 [39]. For CYP2D6 however, individuals with one reduced function and one normal function allele are classified as NMs and CYP2D6 IMs and PMs are defined as activity scores of 0–1.25 and 0, respectively [40]. Here, we followed these current established guidelines and conventions for the translation of genotypes into activity scores and metabolizer phenotypes. As a consequence of these differences in phenotype annotations, we would like to point out to the readers that individuals who carry, for instance, a normal function allele and the reduced function allele CYP2D6*10 (activity score = 0.25), which is highly common across Asia, are classified as NMs, not IMs. Similarly, homozygosity for CYP2D6*10, i.e. a diplotype activity score of 0.5, results in an IM definition, whereas the same activity would be classified as PM in other CYPs.

While well-characterized star alleles play important roles in determining CYP function, a considerable amount of heritable variability in drug response remains unexplained. For instance, elegant twin studies have shown that while 90% of the pharmacokinetic variation of metoprolol and torsemide were heritable, known genetic variants in *CYP2D6* and *CYP2C9* only explained 39% and 2% of the respective variation [41], raising the possibility that additional variants in these or other genes might explain at least part of the “missing heritability”. Further evidence for the potential relevance of as of yet uncharacterized variants in *CYP* genes comes from a retrospective study of 2087 patients taking the antidepressant escitalopram, which revealed that although common *CYP2C19* alleles were well-corelated with escitalopram serum concentrations, substantial variability particularly in the *1/*1 group remained [42]. Our results indicate that, as expected, common star alleles indeed constitute the predominant genetic determinants of functional CYP variability. However, rare, as of yet uncharacterized variants are estimated to account for 1.5–17.5% of the overall genetically encoded functional variability in *CYP* genes. These estimates suggest that the relative contribution of rare variants in *CYPs* is overall lower compared to phase I and phase II enzymes or drug transporters, for which previous studies suggested that approximately 20–40% of the total variability is allotted to rare variants [43–45].

Notably, we here focused on genetic variability in coding regions of the analyzed genes. However, rare variants in regulatory or untranslated regions (UTRs) of *CYP* genes might also modulate gene activity. Previous studies indicated that expression of multiple CYPs, including CYP2C8, CYP2C9 and CYP2C19, was modulated by miRNAs and variants in the respective UTRs might impact these miRNA-mRNA interactions [46]. Furthermore, rare variations in regulatory regions, which are largely understudied due to the challenges associated with their functional interrogation, might contribute substantially to the missing heritability in CYP activity. Importantly, in recent years the repertoire of methodologies and algorithms to predict the functional impact of regulatory variants is rapidly increasing [47]. However, a systematic application of these tools to *CYP* variability in non-coding regions has not been presented and the assessment of the relative importance of such variants for inter-individual variability in drug metabolism remains an important frontier of pharmacogenomic research.

Non-star allele variants that were predicted to be deleterious by APF featured multiple population-specific variants with experimental support of reduced activity. For instance, rs181297724, causing a p.Ala161Pro amino acid exchange in *CYP2C19*, strongly reduced metabolic activity in vitro [48] and was found to be common in Finnish (MAF = 5.6%), whereas it was almost absent in other populations, including Europeans (MAF < 0.1%). In *CYP2D6*, rs79392742 (p.Ala449Asp) was detected in 3.5% of admixed American alleles and was associated with decreased activity. measured with substrates dextromethorphan and metoprolol [49]. Similarly, rs145014075 (p.Ser467X) and rs145308399 (p.Glu97Lys) in *CYP2A6* are common in Ashkenazim and South Asians (MAF = 2.3%), respectively, and have been associated with decreased activity in vitro or in vivo [50, 51]. Notably, the relevance of such variants can differ substantially between populations; for instance, uncharacterized variants in *CYP2C8* and *CYP3A4* are estimated to be as relevant as the characterized star alleles in Asian populations, whereas the contribution of non-star allele variants in *CYP2B6* and *CYP2Cs* in Finnish and Ashkenazi Jewish individuals is very low. These results pinpoint variants and populations in which additional functionally relevant variability in *CYP* genes might be discovered, which might provide useful guidance for the optimization of population-specific genotyping strategies for related drugs.

In summary, the presented analyses provide *CYP* allele frequencies using population-scale sequencing data broadening the data base from previous work by increasing the number of individuals from 56,945 to 141,614. We furthermore make use of improved variant calling quality as well as updated functional annotations and linkage information. In addition, we complement our previous studies by systematically considering rare variants into functional effect predictions in *CYP* genes based on dedicated pharmacogenomic algorithms. The presented data refines the worldwide landscape of ethnogeographic variability in *CYP* genes and emphasizes the importance of considering genetic differences for the optimization of population-specific pharmacotherapy and precision public health.

## Supplementary information

SI Guide

Supplementary Figure 1

Supplementary Table 1

Supplementary Table 2

Supplementary Table 3

## Data Availability

All data were available in the main tables and the supplementary information.
