# Supplementary material for: The genetic landscape of major drug metabolizing cytochrome P450 genes—an updated analysis of population-scale sequencing data
Source: Pharmacogenomics J. 2022 Sep 6;22(5-6):284–93. doi: 10.1038/s41397-022-00288-2 (PMC9674520; doi:10.1038/s41397-022-00288-2)
Supplement: Supplementary file 1 — SI Guide [file 41397_2022_288_MOESM1_ESM.docx]

**Supplementary Material**

**Supplementary Tables**: 3

**Supplementary Figures**: 1

**Supplementary Table 1: Comparisons of common *CYP2B6* and *CYP2D6* variant frequencies between this study and ALFA.**

**Supplementary Table 2: List of all putatively deleterious non-star allele *CYP* variants.**

**Supplementary Table 3: The fraction of genetically encoded functional variability allotted to non-star alleles for all gene-population pairs.**

**Supplementary Figure 1: Global distribution of CYP2C8 phenotypes considering the *CYP2C8*3* allele as functionally neutral.** Numbers indicate the fraction of poor metabolizers (PM; dark red), intermediate metabolizers (IM; light red) and rapid metabolizers (RM; green). The fraction of non-normal metabolizers is indicated by the size of circles on the respective world maps. AFR=African; EAS=East Asian; SAS = South Asian; AMR=admixed Americans; FIN=Finnish; AJ=Ashkenazi Jewish; ME = Middle Eastern; EUR = non-Finnish European.
