# Supplementary figures and images for: The genetic landscape of major drug metabolizing cytochrome P450 genes—an updated analysis of population-scale sequencing data
Source: Pharmacogenomics J. 2022 Sep 6;22(5-6):284–93. doi: 10.1038/s41397-022-00288-2 (PMC9674520; doi:10.1038/s41397-022-00288-2)

# Supplementary Figure 1

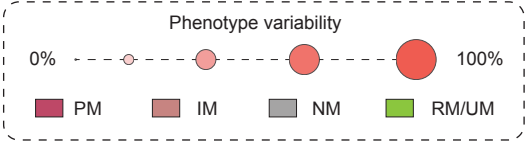

## CYP2C8

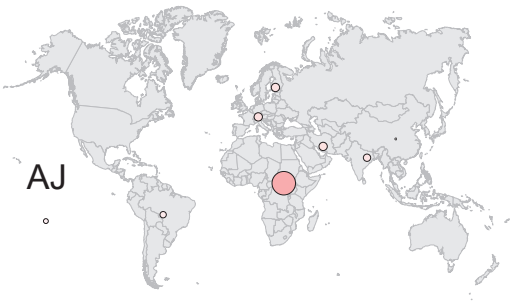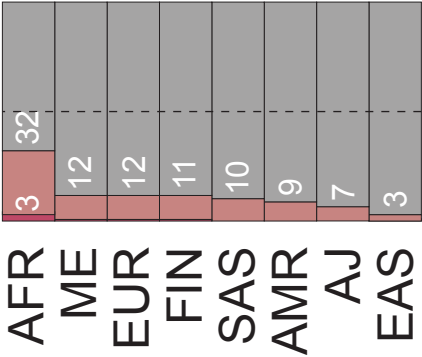

Supplement: Supplementary file 2 — Supplementary Figure 1 [file 41397_2022_288_MOESM2_ESM.pdf]
